# Adsorption Properties and Mechanism of Attapulgite to Graphene Oxide in Aqueous Solution

**DOI:** 10.3390/ijerph19052793

**Published:** 2022-02-27

**Authors:** Na Li, Jiyuan Fang, Ping Jiang, Cuihong Li, Haibo Kang, Wei Wang

**Affiliations:** 1 School of Civil Engineering, Shaoxing University, Shaoxing 312000, China; lina@usx.edu.cn (N.L.); 20020852017@usx.edu.cn (J.F.); jiangping@usx.edu.cn (P.J.); lich@usx.edu.cn (C.L.); 2 School of Civil Engineering, College of Transportation Engineering, Nanjing Tech University, Nanjing 210009, China; nobody@njtech.edu.cn; 3 Department of Civil and Environmental Engineering, National University of Singapore, Singapore 117576, Singapore

**Keywords:** graphene oxide, attapulgite, adsorption isotherm, adsorption thermodynamics, adsorption kinetics

## Abstract

In order to remove toxic graphene oxide (GO) from aqueous solution, attapulgite (ATP) was used as adsorbent to recycle it by adsorption. In this paper, the effects of different pH, adsorbent mass, GO concentration, time and temperature on the adsorption of GO by attapulgite were studied, and the adsorption performance and mechanism were further explored by XRD, AFM, XPS, FTIR, TEM and SEM tests. The results show that when T = 303 K, pH = 3, and the GO concentration is 100 mg/L in 50 mL of aqueous solution, the removal rate of GO by 40 mg of attapulgite reaches 92.83%, and the partition coefficient *K_d_* reaches 16.31. The adsorption kinetics results showed that the adsorption equilibrium was reached at 2160 min, and the adsorption process could be described by the pseudo-second-order adsorption equation, indicating that the adsorption process was accompanied by chemical adsorption and physical adsorption. The isotherm and thermodynamic parameters show that the adsorption of GO by attapulgite is more consistent with the Langmuir isotherm model, and the reaction is a spontaneous endothermic process. The analysis shows that attapulgite is a good material for removing GO, which can provide a reference for the removal of GO in an aqueous environment.

## 1. Introduction

Graphene oxide (GO) is an oxygen-containing graphene derivative. The oxygen-containing groups on the surface of GO easily form composite materials with ions, polymers and other materials, so it has been widely used in the fields of physics, chemistry, biology and materials science [1]. The presence of polar oxygen-containing functional groups on the surface of GO makes it hydrophilic, while the presence of various functional groups such as carboxyl, hydroxyl, and epoxy groups makes its affinity for pollutants in water continuously strengthened, thus it can be used for wastewater treatment [2]. For the wastewater treatment by GO, many scholars have studied it. For example, GO can be used to remove As(III) and cephalexin in aqueous solution. In addition, it can also adsorb metal ions, such as Cr(VI), U(VI), Pb(II), Co(II), etc. [3,4,5,6,7,8].

GO has a strong capacity for wastewater treatment, and often coexists with one or more toxic substances in the aqueous environment, which may lead to more complex forms and toxic effects of composite pollutants, and increase the risk to ecosystems [9]. Adsorbed GO with AS(III) is easily oxidized, which impairs important detoxification pathways in algal cells, thereby exacerbating the toxicity of As(III) to algae [10]. In addition, GO is also toxic to living organisms. Ultra-trace amounts of GO can cause the disappearance of more than 90% of dopamine neurons in zebrafish larvae and the increase of Lewy bodies, which can further lead to Parkinson’s disease-like symptoms and metabolic disorders in zebrafish larvae [11]. The presence of GO increases the accumulation of reactive oxygen species in Drosophila, damages the gut, affects its absorption of nutrients, and ultimately leads to weight loss, slower crawling, stunted growth, and shortened lifespan in Drosophila [12]. The six generations of nematodes were studied in the GO environment, and it was found that due to GO, the development of nematode neurons was defective, the function was easily damaged, and the apoptosis and antioxidant responses were increased [13]. For mammals, GO enters various tissues and organs such as the blood, the gastrointestinal tract, heart, kidney, lung, etc., causing damage to these tissues and organs, resulting in various inflammations, acute allergies and even death [14]. Due to the unique size and morphology of GO, it easily passes through the cell membrane, leading to the destruction of biomolecules such as nucleic acids, lipids and proteins, which in turn leads to DNA damage and induces genotoxicity [15]. Therefore, in view of the popularity of GO and the possibility of leakage, the research on GO adsorption is urgently needed.

By choosing suitable adsorbents, the adsorption process can be a promising technique for GO removal. For example, using goethite or kaolinite as adsorbents can effectively remove graphene oxide from aqueous solutions [16]. As a good adsorbent, clay minerals are widely used to remove heavy metals, antibiotics, dyes, etc. from the aqueous environment [17,18,19]. The adsorption properties of soil benefit from the large surface area, which improves adsorption by promoting ion exchange [20]. Attapulgite is a natural magnesium-aluminosilicate clay that is widely found in all parts of the world. Its main chemical composition is SiO_2_ [21]. Attapulgite not only has good surface area and high surface activity, but studies have shown that GO and attapulgite can be modified and reconstituted by ultrasonic and magnetic stirring methods, and have good adsorption effects on aniline, emulsified oil, propranolol, and Pb(II), U(VI), and plasma [22,23,24,25,26]. However, there are few studies on attapulgite as an adsorbent for the removal of GO in aqueous environments.

In this paper, attapulgite was used as an adsorbent to remove GO in aqueous solution, and the adsorption effect of different pH, temperature, GO concentration, adsorbent mass and different time was studied. At the same time, X-ray diffractometer (XRD), Fourier transform infrared spectroscopy (FTIR), scanning electron microscopy (SEM), Atomic Force Microscopy (AFM), High Resolution Transmission Electron Microscopy (TEM) and X-ray photoelectron spectroscopy (XPS) were used to analyze the microstructure and characterization on the adsorbed precipitates to study and discuss the possible adsorption mechanism. The adsorption process is relatively simple, which is expected to promote the application of attapulgite in GO removal.

## 2. Materials and Methods

### 2.1. Materials

The adsorbate GO used in this experiment was derived from graphene oxide aqueous solution (2 mg/mL), purchased from Suzhou Suzhou Carbon Technology Co., Ltd., Suzhou, China, which is shown in Table 1. Among them, oxidized graphene has a specific surface area of 420 cm^2^/g, and the diameter of the sheet is 5 μm. Attapulgite from China, Jiangsu Province, (Changzhou Dingbang Mine Co., Ltd., Changzhou, China) The specific surface area of the uneven bar is 400 m^2^/g, the hole volume is 0.071 cm^3^/g, the average pore size is 0.51 nm, the glue quality is 55 mL/15 g, and the expandable is 4 mL/g. Its main chemical composition is shown in Table 2.

### 2.2. Characterization

The crystal structure of attapulgite was investigated by X-ray diffraction with CuK_α_ radiation by XRD(Empyrean, Malvern, UK). The functional groups were identified by FTIR(IR Prestigae-21, Shimadzu , TKY, Japan) with a scanning range of 400~4000 cm^−1^. SEM(JSM-6360 LV, JEOL, YKY, Japan), AFM(Dimension Icon, BRUKER, Billerica, MA, USA), TEM(JEM-2100F, JEOL, TKY, Japan) and XPS(Thermo ESCALAB 250XI, Thermo Fisher Scientific, Waltham, MA, USA) were used to measure the morphology and structure before and after adsorption.

### 2.3. Adsorption Test

50 mL of graphene oxide aqueous solution prepared by an appropriate amount of GO and deionized water was poured into a glass bottle. A negligible volume of NaOH was added to adjust the pH of the aqueous solution, and then a pH meter (FE28, METTLER TOLEDO, Columbus, OH, USA) was used to measure and adjust the pH value to between 3 and 10. 

On the basis of referring to previous studies, the amount of adsorbent (10 mg, 20 mg, 30 mg, 40 mg, 50 mg, 60 mg), the initial concentration of GO (40 mg/L, 60 mg/L, 80 mg/L, 100 mg/L, 120 mg/L), and the adsorption effect was studied under pH value (pH3-pH10), temperature (293 K, 303 K, 313 K) and time (0–2880 min) [27]. According to the experimental design, the corresponding mass of attapulgite was added to the graphene oxide aqueous solution.

The glass bottle was then put into a constant temperature shaker and vibrated for 3 h at 240 rpm. After vibration, according to the previous test experience, the glass bottle was put into a thermostat at the set temperature for 24 h curing [28].

After curing, 1 mL of the middle layer supernatant was taken with a pipette gun and diluted to 25 mL with deionized water. After that, the residual GO concentration was measured with an ultraviolet-visible spectrophotometer (UV75N, Shanghai Yoke, Shanghai, China) at a wavelength of 210 nm. According to the initial GO concentration *C*_0_ (mg/L) and the equilibrium concentration *C_e_* (mg/L), the adsorbed amount *Q_e_* (mg/g), the adsorption rate *R*, and the partition coefficient *K_d_* (g/L) were calculated. The calculation formulas are as follows [29]:

(1) $$Q_{e}=\frac{(C_{0}-C_{e})\times V}{m}$$

(2) $$R=\frac{C_{0}-C_{e}}{C_{0}}\times 100\%\;$$

(3) $$K_{d}=\frac{Q_{e}}{C_{e}}\;$$

where *m* (mg) represents the mass of attapulgite, and *V* (mL) represents the volume of the solution. To ensure the accuracy and repeatability of the collected data, all experiments were repeated three times, and the average value of the three experiments was used for subsequent data analysis; error bars were added to the graph to visually understand the degree of dispersion of the experimental data [30].

In order to further study the adsorption behavior, a pseudo-first-order kinetic model and a pseudo-second-order kinetic model are used to fit the adsorption kinetic data. The expression formulas are as follows [31]:

Pseudo-first-order kinetic model:

(4) $$Q_{t}=Q_{e}[1-\exp(-k_{1}t)]\;$$

Pseudo-second-order kinetic model:

(5) $$Q_{t}=\frac{Q_{e}{}^{2}k_{2}t}{1+Q_{e}k_{2}t}$$

where, *Q_e_* represents the adsorption amount at equilibrium, mg/g; *Q_t_* represents the adsorption amount at time *t*, mg/g; *k*_1_ and *k*_2_ are constants, g/(mg·min); t represents the adsorption time, min.

In order to further understand the adsorption mode of attapulgite to GO, the adsorption data were fitted by the Langmuir adsorption isotherm model and the Freundlich adsorption isotherm model, as shown below [32]:

Langmuir adsorption isotherm model:

(6) $$Q_{e}=\frac{K_{L}Q_{max}C_{e}}{1+K_{L}C_{e}}\;$$

Freundlich adsorption isotherm model:

(7) $$Q_{e}=K_{F}C_{e}{}^{\frac{1}{n}}$$

where, *Q_e_* represents the equilibrium adsorption capacity, mg/g; *C_e_* represents the equilibrium concentration, mg/L; *Q_max_* represents the maximum adsorption capacity, mg/g; and *K_L_* , *K_F_* and *n* are constants.

According to the adsorption isotherms at different temperatures, thermodynamic parameters such as standard free energy (∆*G*^0^), enthalpy change (∆*H*^0^) and entropy change (∆*S*^0^) can be calculated by formulas (8)–(10), which are helpful to understand the relationship between the change of temperature and the adsorption process [33].

(8) $$InK_{d}=\frac{\Delta S^{0}}{R}-\frac{\Delta H^{0}}{RT}$$

(9) $$\Delta G^{0}=-RTInK_{d}\;$$

(10) $$K_{d}=\frac{Q_{e}}{C_{e}}\;$$

## 3. Results and Discussion

### 3.1. Morphology Analysis

#### 3.1.1. SEM and TEM Analysis

**Figure 1:**
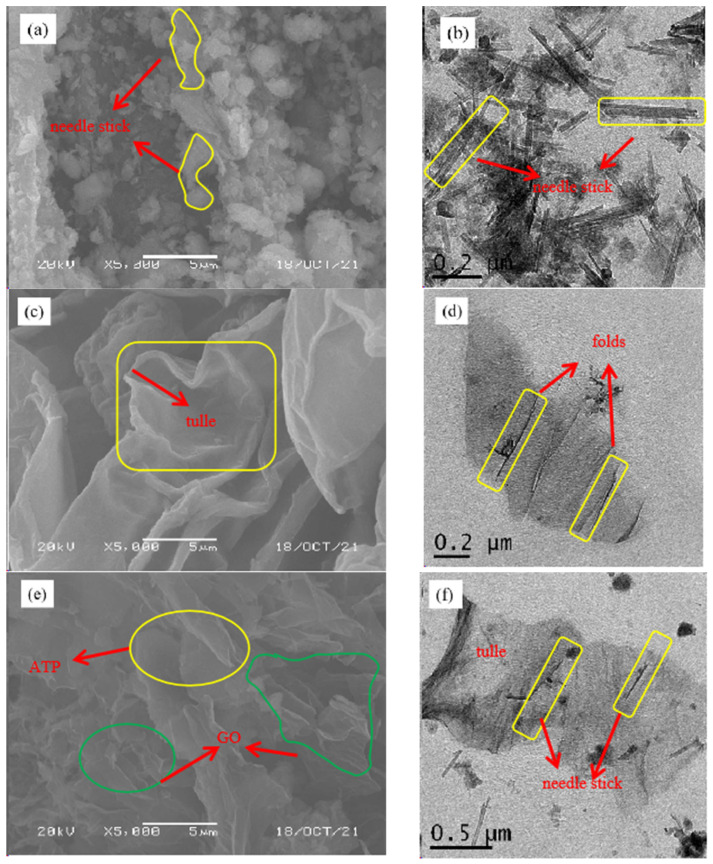
SEM (**a**) and TEM (**b**) of ATP, SEM (**c**) and TEM (**d**) of GO, SEM (**e**) and TEM (**f**) of ATP/GO.

The morphology of the samples before and after adsorption can be observed by SEM and TEM [34], and the results are shown in Figure 1. It can be observed from Figure 1a that attapulgite exhibits agglomeration, which is mainly due to the van der Waals force and hydrogen bonding between attapulgite rod-like crystals [35]. From Figure 1b, it can be seen that attapulgite presents a needle-rod shape, and each rod crystal is closely arranged. From Figure 1c,d, it can be observed that the surface of GO is relatively smooth, showing a gossamer shape and obvious lamellae folding, which is consistent with the research results of Hoor et al. [36]. Figure 1e,f are the images of attapulgite after adsorption of GO. It can be seen from the figures that the needle-shaped attapulgite surface is attached with tulle-like GO, which indicates that GO is adsorbed on the surface of attapulgite. In addition to the study of the microscopic morphology, it is also necessary to further explore the internal structural changes of the samples.

#### 3.1.2. XRD and FTIR Analysis

**Figure 2:**
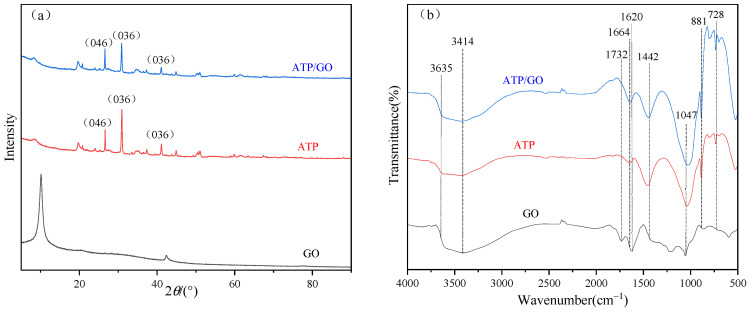
XRD (**a**) and FTIR (**b**) image of GO, ATP, ATP/GO.

In order to further reveal the crystal structure of the sample, XRD can be used to study the substances before and after adsorption n [37], and the results are shown in Figure 2a. It can be clearly observed that GO has a strong diffraction peak near 2θ = 10° [38]. For ATP/GO, the characteristic peak of GO is significantly weakened. By referring to the ICSD standard on the PDF card, 26.62° is marked as SiO_2_ (046), and 30.94° and 41.12° are marked as CaMg(CO_3_)_2_ (036). Comparing ATP with ATP/GO, it was found that the intensity of the strong diffraction peak CaMg(CO_3_)_2_ (036) became weaker, and the change of the diffraction peak indicated that GO was not simply deposited on the surface of attapulgite. 

In addition to XRD, the changes of functional groups before and after the adsorption of GO were analyzed by FTIR [39]. Figure 2b shows the FTIR spectra of GO, ATP and ATP/GO before and after adsorption. Observing GO, the corresponding broad peaks at 3635 cm^−1^ and 3414 cm^−1^ indicate the stretching vibration of O-H and adsorbed H_2_O, 1732 cm^−1^ is the stretching vibration of C=O, 1620 cm^−1^ is the stretching vibration of C=C, 1047 cm^−1^ is the stretching vibration absorption peak of C-O [40,41,42,43]. The functional groups of attapulgite are different from those of GO. There are -OH stretching vibration peaks at 3635 cm^−1^ and 3414 cm^−1^, the bending vibration peaks of adsorbed water in the attapulgite structure at 1664 cm^−1^ and 1460 cm^−1^, and Si-O bonds at 881 cm^−1^ and 728 cm^−1^ [44,45]. Comparing the spectra of GO, ATP and ATP/GO, the image of ATP/GO is similar to that of ATP, and the characteristic peak of oxygen-containing peak of GO gradually weakens or disappears. For example, the peak at 1732 cm^−1^ disappears, and the peak at 1620 cm^−1^ moves to the direction of long wave, indicating that the functional groups of attapulgite are involved in the adsorption of GO [46].

#### 3.1.3. XPS and AFM Analysis

**Figure 3:**
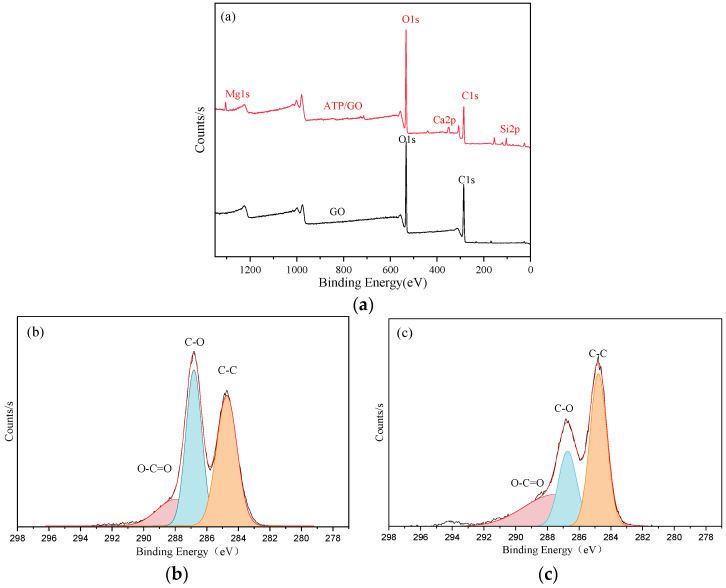
XPS spectra (**a**) of GO and ATP/GO, High C1s deconvolution of GO (**b**) and ATP/GO (**c**).

In order to further explore the adsorption mechanism of GO by attapulgite, XPS can be used to analyze the chemical structure of the material surface to understand the binding energy involved in the interaction between attapulgite and GO [47]. The XPS results of GO and ATP/GO are shown in Figure 3a. It can be observed that there are O1s and C1s in the XPS spectra of GO and ATP/GO. Compared with GO, energy peaks such as Mg1s, Ca2p and Si2p also appears in the energy spectrum of ATP/GO, and the C1s energy peak shows a significant decrease.

Therefore, XPS analysis of GO and ATP/GO can focus on the changes of C1s peaks before and after adsorption. The deconvolution of GO on C1s spectra is mainly divided into three components, approximately 284.8 eV, 286.8 eV and 289.7 eV, corresponding to C-C, C-O and O-C=O, respectively [48]. However, after adsorption, the intensity and position of C1s peak changes, as shown in Figure 3b,c. The peak surface area ratio of C-O decreases from 40.7% to 23.3%, that of O-C=O increases from 15.7% to 33.1%, and the peak position of O-C=O changes from 289.7 eV to 287.9 eV, which shows that the interaction between attapulgite and GO is completed by C-O and O-C=O.

**Figure 4:**
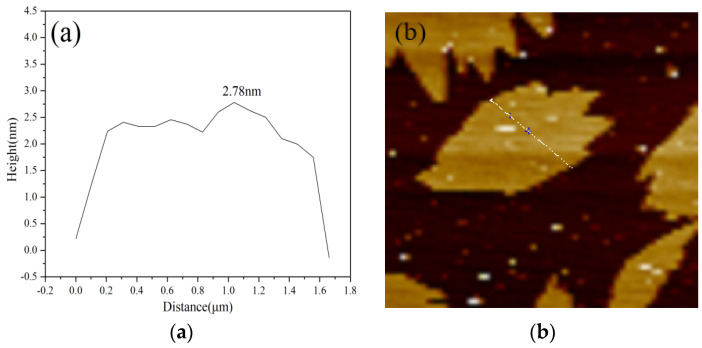

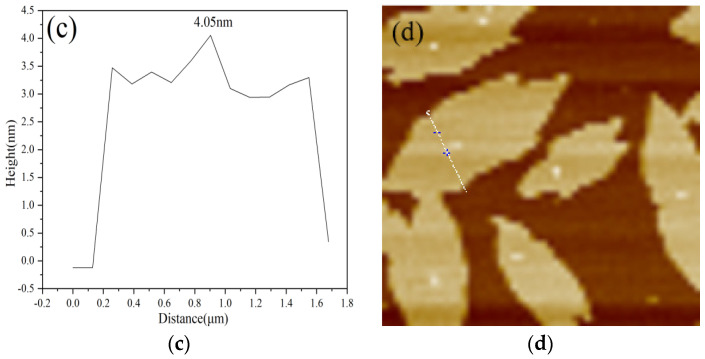
AFM image and the corresponding height profiles of GO (**a**,**b**)and ATP/GO (**c**,**d**).

AFM has atomic-level resolution, which can clearly characterize the changes in morphology and size of samples before and after adsorption [49]. The AFM test results of GO and ATP/GO are shown in Figure 4b,d. Further analysis of the lamellar thickness are shown in Figure 4a,c, and the maximum thicknesses of GO and ATP/GO are 2.78 nm and 4.05 nm, respectively. The thickness of ATP/GO is significantly higher than that of GO, indicating that GO is adsorbed on the surface of attapulgite, which is consistent with the TEM results. Based on the above analysis, it shows that attapulgite can effectively remove GO through the aggregation of GO on the surface of attapulgite.

### 3.2. Effect of PH

**Figure 5:**
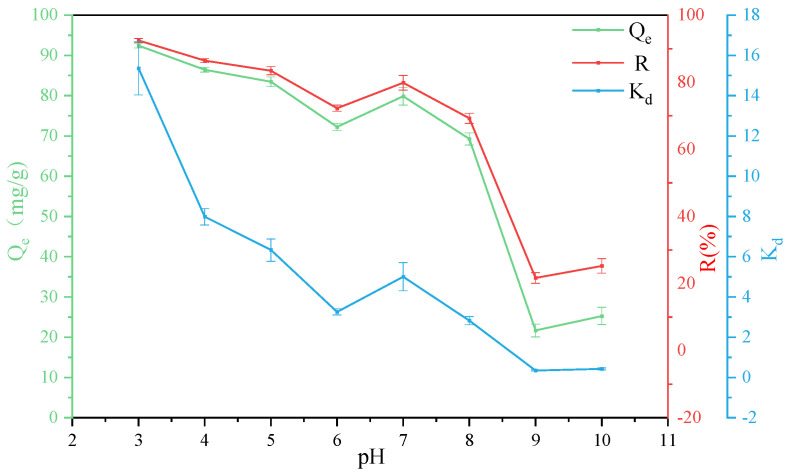
Removal of GO on ATP as a function of pH value.

The change of pH will affect the charge on the surface of the adsorbent, which in turn affects the adsorption effect [50]. In order to study the adsorption ability of attapulgite to GO at different pH, quantitative analysis of GO adsorption amount *Q_e_*, adsorption rate *R*, and partition coefficient *K_d_* was carried out when T = 303 K, the adsorbent mass was 40 mg, and GO concentration was 80 mg/L in 50 mL of the aqueous solution. The calculation results are shown in Figure 5. It can be seen that 92% of GO was adsorbed by attapulgite at pH = 3. The reasons for the better adsorption effect can be mainly divided into the following aspects: on the one hand, GO has a strong self-aggregation force under acidic conditions, and large-scale visible aggregation usually occurs [51]; on the other hand, MgO and CaO in attapulgite will partially dissolve at lower pH to form Mg^2+^ and Ca^2+^ [52]. The existence of Mg^2+^ and Ca^2+^ cations contributes to the compression of the double electric layer and can also penetrate the double electric layer, so as to be directly adsorbed by oxygen-containing functional groups. At the same time, the cations can also interact with large π bonds which ultimately promotes the coagulation of GO [53]. When 3 < pH < 7 and 7 < pH < 10, the adsorption rate decreases continuously, mainly due to the increase of pH, which promotes the deprotonation of the carboxyl group on the GO group, increases the hydrophilicity, and inhibits the binding and accumulation between cations and GO [54]. In addition, due to the large amount of negative charges on the surface of attapulgite and GO, the electrostatic interaction between them is weakened, resulting in a low adsorption capacity of attapulgite to GO [55]. Therefore, lower pH is helpful for the adsorption of GO by attapulgite, and the best adsorption effect occurs when pH = 3.

### 3.3. Effect of Adsorbent Mass

**Figure 6:**
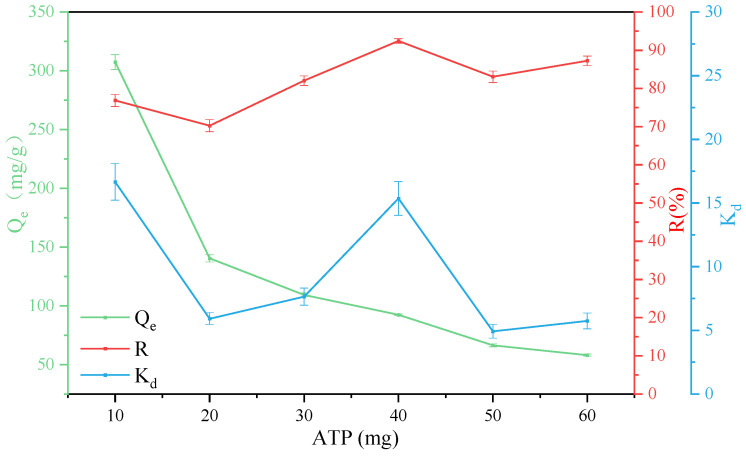
Removal of GO on ATP as a function of ATP contents.

The adsorbent mass also affects the adsorption. Under the conditions of T = 303 K, pH = 3, GO concentration was 80 mg/L, and attapulgite mass was 10 mg, 20 mg, 30 mg, 40 mg, 50 mg and 60 mg, the effects on adsorption capacity, removal rate and partition coefficient were investigated, and the results are shown in Figure 6. With the increase of the attapulgite mass, the removal rate generally shows an upward trend, which is because the number of effective adsorption sites of the adsorbent increases with the increase of the adsorbent mass, thereby improving the adsorption rate and the partition coefficient. However, with the increase of the attapulgite mass, the number of particles per unit volume increases, which is prone to collision and agglomeration, resulting in a decrease in the number of effective active adsorption sites of adsorbent per unit mass, a decrease in the specific surface area of the adsorbent, finally leading to a decrease in the adsorption effect [56]. It can be observed from the figure that attapulgite has the best adsorption effect and relatively low cost when the mass is controlled at 40 mg. Therefore, the attapulgite mass was selected as 40 mg in subsequent studies.

### 3.4. Effect of GO Concentration

**Figure 7:**
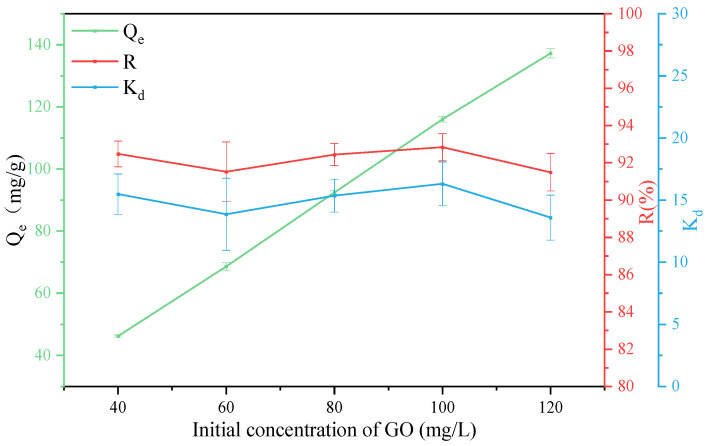
Removal of GO on ATP as a function of GO contents.

In order to explore the effect of GO concentration on the adsorption, under the conditions of T = 303 K, pH = 3, and an attapulgite mass of 40 mg, adsorption tests were carried out on GO solutions with concentration of 40 mg/L, 60 mg/L, 80 mg/L, 100 mg/L and 120 mg/L, respectively, and the test results are shown in Figure 7. It can be seen that with the increase of GO concentration, the adsorption effect increases first and then decreases, which may be because when the GO concentration is low, there are a large number of active adsorption sites on the surface of attapulgite that do not fully function, resulting in a relatively low adsorption rate. When the GO concentration is 100 mg/L, the adsorption rate *R* and the partition coefficient *K_d_* reaches the maximum, which is 92.83% and 16.31, respectively. While the GO concentration continues to increase, although the adsorption amount increases, the adsorption rate decreases, which may be because the increase in GO concentration inhibits the electrostatic interaction between attapulgite and GO.

### 3.5. Adsorption Kinetics

**Figure 8:**
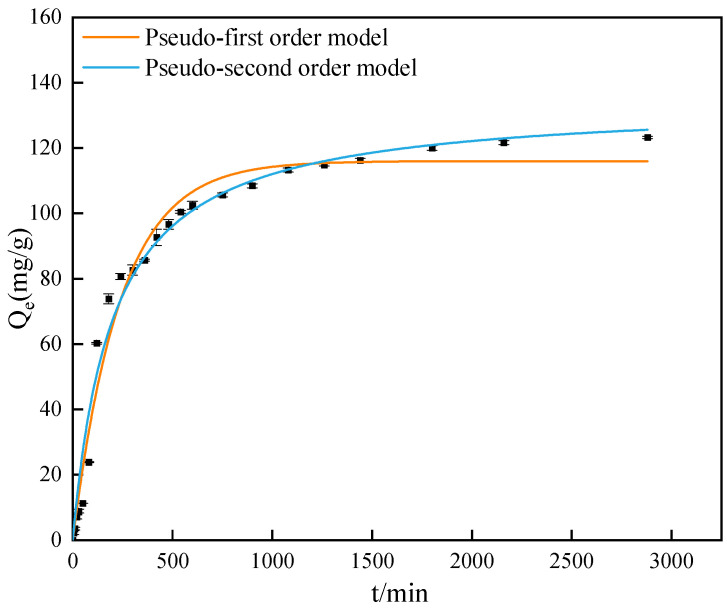
Graph of adsorption capacity over time and Fitting curve of Pseudo-first-order model and Pseudo-second-order model.

The adsorption time also affects the adsorption. The adsorption capacity and adsorption time were studied under the conditions when T = 303 K, pH = 3, GO concentration was 100 mg/L, and the attapulgite mass was 40 mg. The results are shown in Figure 8. It can be seen that the adsorption capacity of attapulgite to GO increases with time and reaches the adsorption equilibrium at 2160 min. In the initial stage of adsorption, the adsorption amount of GO shows a sharp upward trend with the increase of time, and when the time is 480 min, the adsorption amount increases gradually, which is mainly due to the adsorption amount of GO by attapulgite gradually reaching saturation with the passage of adsorption time.

The results are shown in Figure 8 and Table 3. The equilibrium adsorption capacity fitted by the pseudo-first-order kinetic model is 113.664 mg/g, *R*^2^ is 0.980, and that fitted by the pseudo-second-order kinetic model is 130.634 mg/g, *R*^2^ is 0.983. The fitting results of the pseudo-second-order kinetic model are closer to the experimental data, and the correlation coefficient *R*^2^ is relatively higher than that of the pseudo-first-order kinetic model. Therefore, the pseudo-second-order kinetic model is more in line with the kinetic process of GO adsorption by attapulgite, and the adsorption of GO by attapulgite is accompanied by physical adsorption at the same time as the chemical adsorption [57].

### 3.6. Adsorption Isotherm and Thermodynamic Analysis

**Figure 9:**
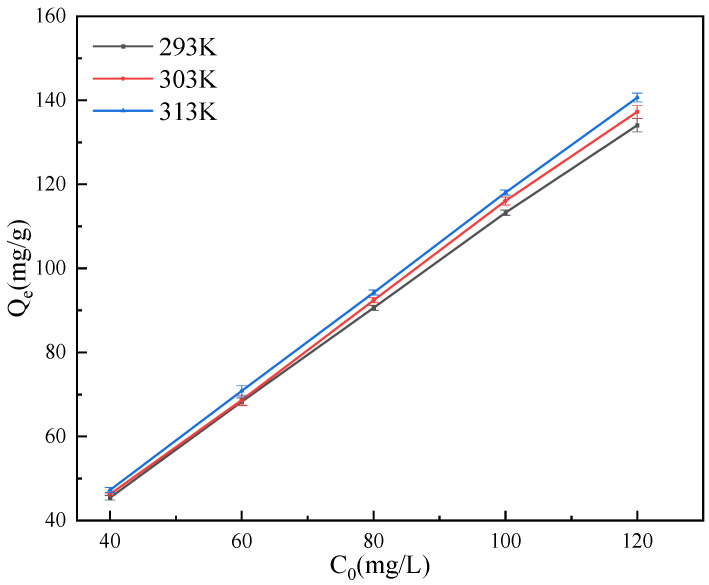
Isotherms of GO adsorption on attapulgite.

The study of adsorption isotherms helps to understand the relationship between adsorbate and adsorbent [58]. In order to further explore the relationship between them, at three different temperatures of 293 K, 303 K and 313 K, adsorption tests were conducted on GO solutions with concentrations of 40 mg/L, 60 mg/L, 80 mg/L, 100 mg/L, and 120 mg/L, when pH = 3, the attapulgite mass was 40 mg. The test results are shown in Figure 9. It can be seen that with the increase of GO concentration, the adsorption capacity of GO by attapulgite is also increasing and increases with the increase of temperature, indicating that the increase of temperature helps to improve the adsorption effect.

**Figure 10:**
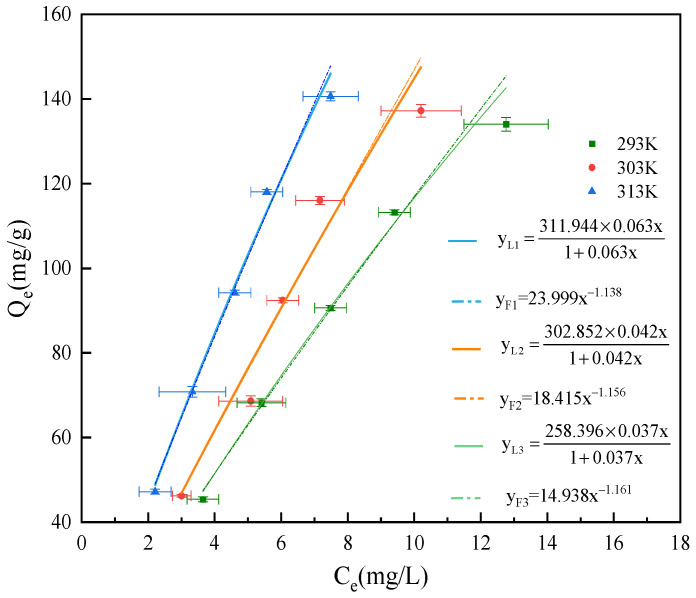
Isothermal equation fitting curve.

In order to further explore the adsorption morphology of GO on the surface of attapulgite, Langmuir and Freundlich adsorption isotherm equations were used to fit the adsorption process of GO on attapulgite. The fitting results are shown in Figure 10 and Table 4. It can be seen from Table 4 that the correlation coefficient *R*^2^ of the fitting results of the Langmuir equation is higher than that of Freundlich equation, indicating that Langmuir equation can more accurately describe the adsorption process of GO by attapulgite, and the adsorption process is based on a uniform monolayer adsorption [59]. In the fitting results of the Langmuir equation, the maximum adsorption capacity *Q_max_* increases with the increase in temperature, indicating that the increase of temperature promotes the progress of the adsorption reaction.

**Figure 11:**
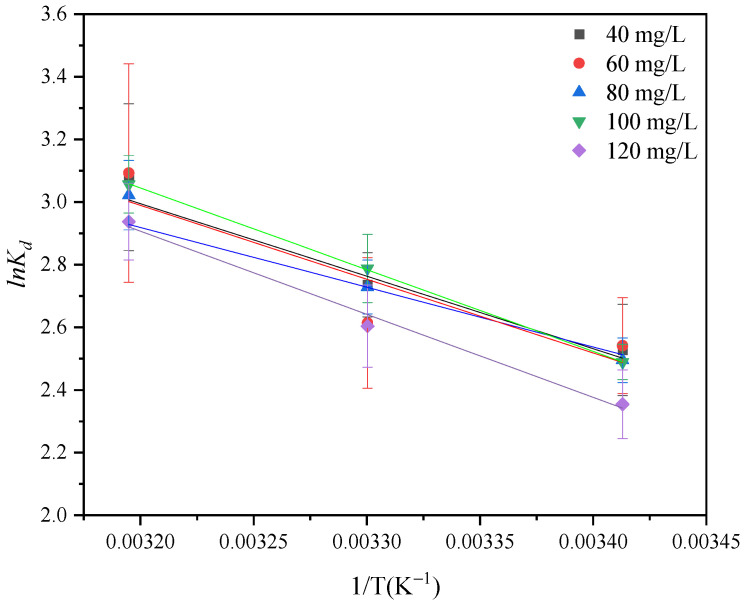
Relationship between *lnK_d_* and 1/T.

In order to analyze the effect of temperature change during the adsorption of GO by attapulgite, the fitting calculation of thermodynamic parameters was performed on the test results. Table 5 and Figure 11 show the thermodynamic fitting curves and calculating parameters. Under the conditions of temperature at 293 K, 303 K, and 313 K, the standard free energy (∆*G*^0^) of GO adsorption by attapulgite at different GO concentrations are all negative, indicating that the adsorption process is spontaneous. With the same concentration, the absolute value of standard free energy (∆*G*^0^) increases with the increase of temperature, indicating that the increase of temperature is conductive to adsorption. The enthalpy change ∆*H*^0^ is positive at different GO concentrations, indicating that the adsorption process is an endothermic reaction, which is consistent with the isotherm fitting results.

In conclusion, the adsorption of GO on attapulgite may be caused by the combined action of multiple factors. From the point of view of electrical properties, both attapulgite and GO are charged, and the electrostatic interaction between them can cause the coagulation of GO [60]. Attapulgite contains Mg^2+^ and Ca^2+^, and the presence of these metal ions also plays a role in the coagulation of GO [61]. Meanwhile, in the coexistence environment of attapulgite and GO, the -OH on the surface of ATP can be complexed with -COOH on the surface of GO [62]. 

## 4. Conclusions

In this paper, the adsorption effect of GO by attapulgite was tested under different conditions, and various characterization methods were used to systematically study its adsorption performance and mechanism.

1. By studying the relationship between pH, adsorbent mass, GO concentration, time and temperature on the adsorption effect, it was found that when T = 303 K, pH = 3, the attapulgite mass is 40 mg, and the GO concentration is 100 mg/L, the adsorption effect is the best, and the removal rate can reach 92.83%. The adsorption effect will increase with the increase of adsorption time. When the adsorption time is 480 min, the increase of adsorption amount tends to be gentle, and the adsorption equilibrium is reached at 2160 min. At the same time, the adsorption capacity of GO by attapulgite increases with the increase of temperature. The higher the concentration is, the more obvious the effect of temperature on the adsorption of GO is.
2. By means of SEM, TEM, XRD, AFM, FTIR and XPS, the materials characterization of GO and ATP/GO was conducted. It was found that GO aggregates on the surface of attapulgite, which is not simple accumulation, but is accompanied by the vibration deformation and interaction of functional groups. Further study of XPS spectra shows that the interaction between GO and attapulgite is mainly completed by C-O and O-C=O.
3. The results of adsorption kinetic studies show that the adsorption of GO by attapulgite is more in line with a pseudo-second-order kinetic equation, and the adsorption process is accompanied by physical adsorption along with chemical adsorption. The results of the isotherm study show that the adsorption of GO by attapulgite is more consistent with the Langmuir isotherm model. Based on the calculation of thermodynamic parameters, the adsorption of GO by attapulgite is an endothermic process.

In summary, attapulgite has a strong adsorption capacity to GO in an aqueous solution, which is helpful for understanding the adsorption behavior of minerals to GO in an aqueous solution by providing a reference for reducing the risk of GO in the aqueous environment. Comparing the adsorption of attapulgite in GO with other materials will help to deepen the understanding of the adsorption of GO by materials. 

The results of other studies are summarized in Table 6 [63,64,65]. Due to factors such as insufficient theoretical knowledge and the limitations of experimental conditions, the study can also explore the adsorption properties of GO on different types of clay minerals. 

## Figures and Tables

**Table 1:** Main element composition of graphene oxide (mass fraction).

Element	C	O	H	S
Content/%	41.70	51.49	2.41	2.00

**Table 2:** Main chemical composition of attapulgite (mass fraction).

Chemical Composition	SiO_2_	MgO	CaO	Al_2_O_3_
Content/%	58.05	11.03	1.18	9.55

**Table 3:** Pseudo-first- and second-order dynamic model fitting parameters.

pH	Temperature(K)	Pseudo-First-Order Model			Pseudo-Second-Order Model		
		*Q_e_* (mg/g)	*k*_1_ g/(mg·min)	*R* ^2^	*Q_e_* (mg/g)	*k*_2_ g/(mg·min)	*R* ^2^
3	303 k	113.664	0.004	0.980	130.634	3.758 × 10^−5^	0.983

**Table 4:** Adsorption isotherm equation fitting parameters.

*C*_0_ (mg/L)	pH	Temperature(K)	Langmuir			Freundlich		
			*Q_max_* (mg/g)	*K_L_* (L/mg)	*R* ^2^	*K_F_* (mg/g)	*n*	*R* ^2^
100	3	313	311.944	0.063	0.993	23.999	1.138	0.987
		303	302.852	0.042	0.984	18.415	1.156	0.978
		293	258.396	0.037	0.996	14.938	1.161	0.986

**Table 5:** Thermodynamic Fitting Parameters.

*C*_0_ (mg/L)	Δ*G*^0^/(kJ·mol^−1^)			Δ*H*^0^/(kJ·mol^−1^)	Δ*S*^0^/(J·mol^−1^·K^−1^)
	293 K	303 K	313 K		
40	−6.159	−6.892	−8.014	19.248	86.499
60	−6.192	−6.586	−8.048	15.883	75.100
80	−6.079	−6.875	−7.864	19.598	87.571
100	−6.064	−7.024	−7.955	21.681	94.696
120	−5.736	−6.559	−7.644	22.056	94.746

**Table 6:** Adsorbate Adsorption of GO Comparison.

Adsorbent	Layered Double Hydroxide	Calcareous Sand	Iron Tailings	Attapulgite
Adsorbent dosage (mg)	5, 10, 15, 20, 25	30, 40, 50, 60, 70	30, 40, 50, 60, 70	10, 20, 30, 40, 50, 60
GO initial concentration (mg/L)	20, 40, 60, 80, 100, 120	80, 100, 120, 140, 160	40, 60, 80, 100	40, 60, 80, 100, 120, 140, 160
pH effect	Alkaline environment inhibits adsorption	Alkaline environment inhibits adsorption	pH = 7 is the best	pH = 3 is the best
Optimal removal rate	92%	91.5%	85.92%	92.83%
Equilibrium time (min)	360 min	300 min	1680 min	2160 min
References	[63]	[64]	[65]	This study

## Data Availability

Not applicable.

## References

[1] Huang X.M., Liu L.Z., Zhou S., Zhao J.J. (2020). Physical properties and device applications of graphene oxide. Front. Phys..

[2] Velusamy S., Roy A., Sundaram S., Mallick T.K. (2021). A Review on Heavy Metal Ions and Containing Dyes Removal Through Graphene Oxide-Based Adsorption Strategies for Textile Wastewater Treatment. Chem. Rec..

[3] Reynosa-Martinez A.C., Tovar G.N., Gallegos W.R., Rodriguez-Melendez H., Torres-Cadena R., Mondragon-Solorzano G., Barroso-Flores J., Alvarez-Lemus M.A., Montalvo V.G., Lopez-Honorato E. (2020). Effect of the degree of oxidation of graphene oxide on As(III) adsorption. J. Hazard. Mater..

[4] Wernke G., Shimabuku-Biadola Q.L., dos Santos T.R.T., Silva M.F., Fagundes-Klen M.R., Bergamasco R. (2020). Adsorption of cephalexin in aqueous media by graphene oxide: Kinetics, isotherm, and thermodynamics. Environ. Sci. Pollut. Res..

[5] Mondal N.K., Chakraborty S. (2020). Adsorption of Cr(VI) from aqueous solution on graphene oxide (GO) prepared from graphite: Equilibrium, kinetic and thermodynamic studies. Appl. Water Sci..

[6] Liu X., Sun J., Xu X.T., Alsaedi A., Hayat T., Li J.X. (2019). Adsorption and desorption of U(VI) on different-size graphene oxide. Chem. Eng. J..

[7] Zhang J.F., Xie X.D., Meng X.G., Li Y., Zhu W.H. (2020). The critical role of oxidative debris in the adsorption and desorption of Pb(II) to graphene oxides under alkaline groundwater conditions. Sci. Total Environ..

[8] Lingamdinne L.P., Koduru J.R., Roh H., Choi Y.L., Chang Y.Y., Yang J.K. (2016). Adsorption removal of Co(II) from waste-water using graphene oxide. Hydrometallurgy.

[9] Chowdhury I., Duch M.C., Mansukhani N.D., Hersam M.C., Bouchard D. (2013). Colloidal Properties and Stability of Graphene Oxide Nanomaterials in the Aquatic Environment. Environ. Sci. Technol..

[10] Cao X., Ma C., Zhao J., Musante C., White J.C., Wang Z., Xing B. (2019). Interaction of graphene oxide with co-existing arsenite and arsenate: Adsorption, transformation and combined toxicity. Environ. Int..

[11] Ren C.X., Hu X.G., Li X.Y., Zhou Q.X. (2016). Ultra-trace graphene oxide in a water environment triggers Parkinson’s disease-like symptoms and metabolic disturbance in zebrafish larvae. Biomaterials.

[12] Guo Q., Yang Y., Zhao L., Chen J., Duan G., Yang Z., Zhou R. (2022). Graphene oxide toxicity in W1118 flies. Sci. Total Environ..

[13] Jin L., Dou T.-T., Chen J.-Y., Duan M.-X., Zhen Q., Wu H.-Z., Zhao Y.-L. (2022). Sublethal toxicity of graphene oxide in Caenorhabditis elegans under multi-generational exposure. Ecotoxicol. Environ. Saf..

[14] Lin Y.F., Zhang Y., Li J., Kong H.T., Yan Q.L., Zhang J.C., Li W., Ren N., Cui Y.Z., Zhang T. (2020). Blood exposure to graphene oxide may cause anaphylactic death in non-human primates. Nano Today.

[15] Ou L.L., Lv X.J., Wu Z.X., Xia W.B., Huang Y.D., Chen L.Y., Sun W.J., Qi Y., Yang M., Qi L. (2021). Oxygen content-related DNA damage of graphene oxide on human retinal pigment epithelium cells. J. Mater. Sci. Mater. Med..

[16] Liu X., Sun J., Xu X., Sheng G., Sun Y., Huang Y., Alsaedi A., Hayat T., Li J. (2019). Is the interaction between graphene oxide and minerals reversible?. Environ. Pollut..

[17] Huang B., Yuan Z.J., Li D.Q., Zheng M.G., Nie X.D., Liao Y.S. (2020). Effects of soil particle size on the adsorption, distribution, and migration behaviors of heavy metal(loid)s in soil: A review. Environ. Sci. Processes Impacts.

[18] Conde-Cid M., Fernandez-Calvino D., Nunez-Delgado A., Fernandez-Sanjurjo M.J., Arias-Estevez M., Alvarez-Rodriguez E. (2020). Estimation of adsorption/desorption Freundlich’s affinity coefficients for oxytetracycline and chlortetracycline from soil properties: Experimental data and pedotransfer functions. Ecotoxicol. Environ. Saf..

[19] Jedidi A., Kraiem A., Dardouri S., Marcoux M., Sghaier J. (2020). Adsorption of Dye on a Tunisian Unsaturated Layered Soil: Physical and Numerical Modeling. Eurasian Soil Sci..

[20] Otunola B.O., Ololade O.O. (2020). A review on the application of clay minerals as heavy metal adsorbents for remediation purposes. Environ. Technol. Innov..

[21] Yan Z., Liu Q., Liang L., Ouyang J. (2021). Surface hydroxyls mediated CO_2_ methanation at ambient pressure over attapulgite-loaded Ni-TiO_2_ composite catalysts with high activity and reuse ability. J. CO_2_ Util..

[22] Deng Q.L., Chen C., Lei Q., Liang J.H., Zhang T.H., Jiang J.L. (2018). Adsorption of aniline from aqueous solution using graphene oxide-modified attapulgite composites. RSC Adv..

[23] Liu J., Liu J., Zhong J.P., Shen J.L., Ren S.L. (2021). Preparation of Graphene Oxide/Attapulgite Composites and Their Demulsification Performance for Oil-in-Water Emulsion. Energ. Fuel.

[24] Deng Y.H., Li Y.N., Nie W.J., Gao X., Zhang L., Yang P.L., Tan X.C. (2019). Fast Removal of Propranolol from Water by Attapulgite/Graphene Oxide Magnetic Ternary Composites. Materials.

[25] Wei B.G., Cheng X.B., Wang G., Li H., Song X.S., Dai L. (2019). Graphene Oxide Adsorption Enhanced by Attapulgite to Remove Pb (II) from Aqueous Solution. Appl. Sci..

[26] Liu X., Xu X.T., Sun J., Alsaedi A., Hayat T., Li J.X., Wang X.K. (2018). Insight into the impact of interaction between attapulgite and graphene oxide on the adsorption of U(VI). Chem. Eng. J..

[27] Abba M.U., Che Man H., Azis S., Idris A.I., Hazwan Hamzah M., Abdulsalam M. (2021). Synthesis of nano-magnetite from industrial mill chips for the application of boron removal: Characterization and adsorption efficacy. Int. J. Environ. Res. Public Health.

[28] Piaskowski K., Zarzycki P.K. (2020). Carbon-based nanomaterials as promising material for wastewater treatment processes. Int. J. Environ. Res. Public Health.

[29] Liu J., Zhang J., Xing L., Wang D., Wang L., Xiao H., Ke J. (2021). Magnetic Fe_3_O_4_/attapulgite hybrids for Cd (II) adsorption: Performance, mechanism and recovery. J. Hazard. Mater..

[30] Huang R.L., Lin Q.T., Zhong Q.F., Zhang X.F., Wen X.Q., Luo H.Y. (2020). Removal of Cd(II) and Pb(II) from aqueous solution by modified attapulgite clay. Arab. J. Chem..

[31] Simonin J.-P. (2016). On the comparison of pseudo-first order and pseudo-second order rate laws in the modeling of adsorption kinetics. Chem. Eng. J..

[32] Neolaka Y.A.B., Lawa Y., Naat J.N., Riwu A.A.P., Iqbal M., Darmokoesoemo H., Kusuma H.S. (2020). The adsorption of Cr(VI) from water samples using graphene oxide-magnetic (GO-Fe_3_O_4_) synthesized from natural cellulose-based graphite (kusambi wood or Schleichera oleosa): Study of kinetics, isotherms and thermodynamics. J. Mater. Res. Technol..

[33] Fernandes E.P., Silva T.S., Carvalho C.M., Selvasembian R., Chaukura N., Oliveira L.M., Meneghetti S.M.P., Meili L. (2021). Efficient adsorption of dyes by γ-alumina synthesized from aluminum wastes: Kinetics, isotherms, thermodynamics and toxicity assessment. J. Environ. Chem. Eng..

[34] Abou Taleb M.F., Abou El Fadl F.I., Albalwi H. (2021). Adsorption of Toxic dye in wastewater onto Magnetic NVP/CS Nanocomposite hydrogels synthesized using gamma radiation. Sep. Purif. Technol..

[35] Cui M.K., Mu P., Shen Y.Q., Zhu G.R., Luo L., Li J. (2020). Three-dimensional attapulgite with sandwich-like architecture used for multifunctional water remediation. Sep. Purif. Technol..

[36] Hoor Y.Q., Au P.I., Mubarak N.M., Khalid M., Jagadish P., Walvekar R., Abdullah E.C. (2020). Surface force arising from Adsorbed graphene oxide in kaolinite suspensions. Colloid Surf. A.

[37] Wang M., Guo Y., Fu X., Cui H., Sun T., Tang Y., Liu Q. (2021). Facile synthesis of novel Zn_3_ (OH) _2_V_2_O_7_· 2H_2_O nanocables with excellent adsorption properties. Mater. Lett..

[38] Yang Z., Liu X., Liu X., Wu J., Zhu X., Bai Z., Yu Z. (2021). Biointerfaces, S.B. Preparation of β-cyclodextrin/graphene oxide and its adsorption properties for methylene blue. Colloid Surf. B Biointerfaces.

[39] Li Q., Zhao S., Wang Y. (2021). Mechanism of Oxytetracycline Removal by Coconut Shell Biochar Loaded with Nano-Zero-Valent Iron. Int. J. Env. Res. Pub. Heal..

[40] Khalil W.F., El-Sayyad G.S., El Rouby W.M.A., Sadek M.A., Farghali A.A., El-Batal A.I. (2020). Graphene oxide-based nanocomposites (GO-chitosan and GO-EDTA) for outstanding antimicrobial potential against some Candida species and pathogenic bacteria. Int. J. Biol. Macromol..

[41] Boukhoubza I., Khenfouch M., Achehboune M., Leontie L., Galca A.C., Enculescu M., Carlescu A., Guerboub M., Mothudi B.M., Jorio A. (2020). Graphene Oxide Concentration Effect on the Optoelectronic Properties of ZnO/GO Nanocomposites. Nanomaterials.

[42] Ma Y.J., Ye Y.P., Wan H.Q., Chen L., Zhou H.D., Chen J.M. (2020). Chemical modification of graphene oxide to reinforce the corrosion protection performance of UV-curable polyurethane acrylate coating. Prog. Org. Coat..

[43] Wu Q., He J.Q., Wang F., Yang X., Zhu J.F. (2020). Comparative study on effects of covalent-covalent, covalent-ionic and ionicionic bonding of carbon fibers with polyether amine/GO on the interfacial adhesion of epoxy composites. Appl. Surf. Sci..

[44] Meng F.Y., Song M., Chen Y.Y., Wei Y.X., Song B., Cao Q.Q. (2021). Promoting adsorption of organic pollutants via tailoring surface physicochemical properties of biomass-derived carbon-attapulgite. Environ. Sci. Pollut. Res..

[45] Song S., Liu Z., Zhang J., Jiao C.Z., Ding L., Yang S.R. (2020). Synthesis and Adsorption Properties of Novel Bacterial Cellulose/Graphene Oxide/Attapulgite Materials for Cu and Pb Ions in Aqueous Solutions. Materials.

[46] Wang C.Y., Zeng W.J., Jiang T.T., Chen X., Zhang X.L. (2019). Incorporating attapulgite nanorods into graphene oxide nanofiltration membranes for efficient dyes wastewater treatment. Sep. Purif. Technol..

[47] Wang R.-s., Li Y., Shuai X.-x., Liang R.-h., Chen J., Liu C.M. (2021). Pectin/activated carbon-based porous microsphere for Pb^2+^ adsorption: Characterization and adsorption behaviour. Polymers.

[48] Al-Gaashani R., Najjar A., Zakaria Y., Mansour S., Atieh M.A. (2019). XPS and structural studies of high quality graphene oxide and reduced graphene oxide prepared by different chemical oxidation methods. Ceram. Int..

[49] Chimonyo W., Fletcher B., Peng Y.J.M.E. (2021). Adsorption and morphology of oxidized starches on graphite. Miner. Eng..

[50] Belhaj A.F., Elraies K.A., Mahmood S.M., Zulkifli N.N., Akbari S., Hussien O.S. (2020). The effect of surfactant concentration, salinity, temperature, and pH on surfactant adsorption for chemical enhanced oil recovery: A review. J. Pet. Explor. Prod. Technol..

[51] Shih C.J., Lin S.C., Sharma R., Strano M.S., Blankschtein D. (2012). Understanding the pH-Dependent Behavior of Graphene Oxide Aqueous Solutions: A Comparative Experimental and Molecular Dynamics Simulation Study. Langmuir.

[52] Jiang P., Zhou L., Wang W., Li N., Zhang F. (2022). Performance and mechanisms of fly ash for graphene oxide removal from aqueous solution. Environ. Sci. Pollut. Res..

[53] Tang H., Zhang S.Y., Huang T.L., Cui F.Y., Xing B.S. (2020). Effects of pH and electrolytes on the sheet-to-sheet aggregation mode of graphene oxide in aqueous solutions. Environ. Sci.-Nano.

[54] Zhao L., Yang S.-T., Feng S., Ma Q., Peng X., Wu D. (2017). Preparation and application of carboxylated graphene oxide sponge in dye removal. Int. J. Env. Res. Public Health.

[55] Nan F., Liu C.B., Pu J.B. (2019). Anticorrosive performance of waterborne epoxy coatings containing attapulgite/graphene nanocomposites. Surf. Topogr. Metrol. Prop..

[56] Benjelloun M., Miyah Y., Evrendilek G.A., Zerrouq F., Lairini S. (2021). Recent Advances in Adsorption Kinetic Models: Their Application to Dye Types. Arab. J. Chem..

[57] Salis A., Meloni D., Ligas S., Casula M.F., Monduzzi M., Solinas V., Dumitriu E. (2005). Physical and chemical adsorption of Mucor javanicus lipase on SBA-15 mesoporous silica. Synthesis, structural characterization, and activity performance. Langmuir.

[58] Sharma S., Sharma G., Kumar A., AlGarni T.S., Naushad M., ALOthman Z.A., Stadler F.J. (2022). Adsorption of cationic dyes onto carrageenan and itaconic acid-based superabsorbent hydrogel: Synthesis, characterization and isotherm analysis. J. Hazard. Mater..

[59] Wang Z.J., Jia Y.N., Song W.K., Li X.Q., Xu K., Wang Z. (2021). Optimization of boron adsorption from desalinated seawater onto UiO-66-NH2/GO composite adsorbent using response surface methodology. J. Clean. Prod..

[60] Han Y., Wang L., Guo X., Jiao T., Ding H. (2021). Enhanced adsorption efficiency of graphene oxide by electrostatic field for Hg (II) removal from water. J. Mol. Liq..

[61] Kong Q., Preis S., Li L., Luo P., Wei C., Li Z., Hu Y., Wei C. (2020). Relations between metal ion characteristics and adsorption performance of graphene oxide: A comprehensive experimental and theoretical study. Sep. Purif. Technol..

[62] Kumar V., Bahadur N., Sachdev D., Gupta S., Reddy G., Pasricha R. (2014). Restructural confirmation and photocatalytic applications of graphene oxide–gold composites synthesized by Langmuir–Blodgett method. Carbon.

[63] Kang H., Gu J., Liu G., Li B., Wang W. (2021). Performance and mechanism of layered double hydroxide to remove graphene oxide in aqueous solution. Nat. Environ. Pollut. Technol..

[64] Lv B., Yu W., Luo J., Qian B., Asefa M.B., Li N. (2021). Study on the Adsorption Mechanism of Graphene Oxide by Calcareous Sand in South China Sea. Adsorpt. Sci. Technol..

[65] Zhou J., Yao L., Wang Y., Zhao W., Gu J. (2021). Study on the adsorption properties of iron tailings for GO. Coatings.

